# Vacation

**Published:** 1897-08

**Authors:** 


### Editorial.

### Vacation.

The time of the year has again come around when many people think it their duty to take a vacation. Some because they really need it, or imagine they do, others from habit, and still others because it is fashionable.

This custom is perhaps indulged in by as large a proportion of dentists as any other class of people.

The reason usually assigned for a vacation is to recuperate lost energy and strength, and to have diversion from the exhausting labor and confinement incident to professional business. It is true that in many instances recuperation is much needed, in others the need is only imaginary. Oftentimes the physical exhaustion is greater during the vacation than at any other equal period during the year.

The method of renewing lost strength and energy, and it may be health, is by rest; quiet and the observance of general hygieanic requirements together with gentle, health-giving exercise and occupation for mind and body. It is not as a rule obtained by much wearisome travel from one locality to another, nor by the excessive changes in methods of living that are so generally indulged during vacation times. It often occurs that recuperation is as absolutely required after a vacation, as at its beginning.

Now, let it not be understood that we would discountenance or condemn vacations, but the question arises, may not the usual vacation custom be so modified as to more certainly secure the desired results and avoid the objectionable features?

These remarks are made to open the way for suggesting possibly a better way.

Now, it is true that in our large cities, and many smaller places also, the practice of dentistry as usually conducted, is very exhausting and as followed in many instances, within a few years causes to a greater or lesser degree a breakdown of physical strength and energy. The confinement in an office as we usually find it, of from nine to ten hours per day, is a great violation of health laws, and when is added to this, the severe labor and nervous strain to which a large number in the profession subject themselves, it is not a matter of surprise that health impaired and exhaustion more or less pronounced are result. Now what remedy shall be suggested? First, let there be less ambition to establish and conduct a large professional business. Second, let the office so far as light, heat, air and cleanliness and all office conditions, be as nearly perfect as possible. Have every equipment of the office so perfect in system and arrangement, that it will occasion no annoyance to the dentist in any particular. Indeed, the dentist should have his whole environment as free from annoyance and irritation as it is possible to make it. His business, family and social affairs should be of such a character, and so conducted as to cause him the least possible amount of disturbance or anxiety. In regard to the work or labor in the practice of dentistry, it should usually be suspended whenever it becomes fatigueing or exhausting, or when any marked nervous irritation occurs. How can these embarrassments be avoided? It may be answered by avoiding the continuous and persistent labor so frequently indulged. What's the rule? In answer to this question it may be said, no rule of universal application can be given; every dentist in this respect must be a law unto himself, here as in every other occupation each dentist should have his own rule. The strong and vigorous may labor from seven to nine hours per day while another should restrict himself to three or four hours, according to the indications which are plainly within his comprehension.

If a dentist cannot work two hours a day comfortably and without fatigue, he should abandon the practice for some occupation better suited to his condition. No man should engage in the practice of dentistry who has not a love, or at least a liking for it. If we practice but three or four hours per day how shall the rest of the time be employed? To this a definite answer, of universal application, cannot be given. In some cases it is best to devote this time to rest, or to some pleasant, congenial and health-giving occupation. For this a great many things might be suggested but it is not necessary here to specify, as any one of a good degree of mental resource will be able to decide for himself.

If the suggestions here made are well followed the ordinary vacation season with its accompaniments would be much less a matter of necessity, and breaking down of health and strength much less frequent.

